# Melatonin therapy to improve nocturnal sleep in critically ill patients: encouraging results from a small randomised controlled trial

**DOI:** 10.1186/cc6871

**Published:** 2008-04-18

**Authors:** Richard S Bourne, Gary H Mills, Cosetta Minelli

**Affiliations:** 1 Sheffield Teaching Hospitals, Critical Care Department, Northern General Hospital, Herries Road, Sheffield, UK, S5 7AU; 2 Sheffield Teaching Hospitals, Critical Care Directorate, Royal Hallamshire Hospital, Glossop Road, Sheffield, UK, S10 2JF; 3 Respiratory Epidemiology and Public Health Group, National Heart and Lung Institute, Imperial College London, Emmanuel Kaye Building, Manresa Road, London, UK, SW3 6LR

## Abstract

**Introduction:**

Sleep disturbances are common in critically ill patients and when sleep does occur it traverses the day-night periods. The reduction in plasma melatonin levels and loss of circadian rhythm observed in critically ill patients receiving mechanical ventilation may contribute to this irregular sleep-wake pattern. We sought to evaluate the effect of exogenous melatonin on nocturnal sleep quantity in these patients and, furthermore, to describe the kinetics of melatonin after oral administration in this patient population, thereby guiding future dosing schedules.

**Methods:**

We conducted a randomised double-blind placebo-controlled trial in 24 patients who had undergone a tracheostomy to aid weaning from mechanical ventilation. Oral melatonin 10 mg or placebo was administered at 9 p.m. for four nights. Nocturnal sleep was monitored using the bispectral index (BIS) and was expressed in terms of sleep efficiency index (SEI) and area under the curve (AUC). Secondary endpoints were SEI measured by actigraphy and nurse and patient assessments. Plasma melatonin concentrations were measured in nine patients in the melatonin group on the first night.

**Results:**

Nocturnal sleep time was 2.5 hours in the placebo group (mean SEI = 0.26, 95% confidence interval [CI] 0.17 to 0.36). Melatonin use was associated with a 1-hour increase in nocturnal sleep (SEI difference = 0.12, 95% CI -0.02 to 0.27; *P *= 0.09) and a decrease in BIS AUC indicating 'better' sleep (AUC difference = -54.23, 95% CI -104.47 to -3.98; *P *= 0.04). Results from the additional sleep measurement methods were inconclusive. Melatonin appeared to be rapidly absorbed from the oral solution, producing higher plasma concentrations relative to similar doses reported in healthy individuals. Plasma concentrations declined biexponentially, but morning (8 a.m.) plasma levels remained supraphysiological.

**Conclusion:**

In our patients, nocturnal sleep quantity was severely compromised and melatonin use was associated with increased nocturnal sleep efficiency. Although these promising findings need to be confirmed by a larger randomised clinical trial, they do suggest a possible future role for melatonin in the routine care of critically ill patients. Our pharmacokinetic analysis suggests that the 10-mg dose used in this study is too high in these patients and may lead to carryover of effects into the next morning. Reduced doses of 1 to 2 mg could be used in future studies.

**Trial registration:**

Current Controlled Trials ISRCTN47578325.

## Introduction

Sleep disturbances are common in critically ill patients, who present a loss of monophasic nocturnal sleep combined with frequent diurnal naps (irregular sleep-wake pattern) [1] as well as a reduction in deeper, more restorative phases such as slow-wave sleep (SWS) and rapid eye movement (REM) sleep [2]. Although the consequences of such prolonged sleep fragmentation are unknown, they may be comparable to the significant morbidity associated with prolonged sleep deprivation [3]. Patients themselves perceive sleep disturbances to be one of the most stressful components of their intensive care stay [4].

Nocturnal secretion of melatonin synchronises the sleep-wake and dark-light cycles [5], and disruption to the normal timing and amplitude of the circadian rhythm of melatonin secretion is associated with reduced sleep [6,7]. Reduction in plasma melatonin levels and lack of circadian rhythm have been shown in critical care patients undergoing mechanical ventilation [8-11].

Exogenous melatonin has been demonstrated to be safe and effective in the treatment of other circadian rhythm sleep disorders [12]. This study aimed to examine the effect of exogenous melatonin on nocturnal sleep in patients being weaned from mechanical ventilation. The optimum oral dose to use in this population is also unknown and therefore a pharmacokinetic analysis of plasma melatonin concentrations was also undertaken.

## Materials and methods

We conducted a randomised double-blind placebo-controlled trial in patients admitted to an adult general intensive care unit (ICU) with acute respiratory failure requiring mechanical ventilation and tracheostomy to assist weaning. Exclusion criteria were an expected ICU length of stay of less than 5 days, pre-admission treatment of sleep disturbances, contraindications to enteral feeding, a history of convulsions, psychiatric or neurological disease, alcohol consumption of greater than or equal to 50 units per week or drug use, sleep apnoea, severe heart failure (New York Heart Association classification III/IV), and low levels of consciousness, defined as values of below 4 on the Sedation Agitation Scale (SAS) [13]. The local ethics committee approved the study protocol and all patients provided written informed consent.

Patients were randomly assigned to melatonin or placebo by the pharmacy, using random assignment in blocks of four. Melatonin 10 mg, formulated in an oral liquid, or matching placebo was administered enterally at 9 p.m. for four consecutive nights [14]. Propofol and alfentanil were discontinued at least 30 hours, and morphine and midazolam at least 48 hours, before study entry. No hypnotics were allowed during the study. Haloperidol was allowed in very agitated patients (SAS of greater than or equal to 6). Earplugs and eye masks were made available for use at the patients' discretion, and staff meetings and posters were employed to encourage staff to minimise environmental, nursing, and clinical disturbances during the nocturnal study periods. Environmental disturbances were documented based on a locally derived scale composed of light interruptions, clinical activities, and use of invasive instrumentation (Additional file 1). The nurses also subjectively ranked the noise level each night (Additional file 1). Baseline nocturnal illuminance at the head of each patient bed when all lights were off was recorded using a light meter (Luxmeter PU150; Eagle International, Wembley, UK). Drug records were compiled daily for drugs known to adversely affect sleep [15] or melatonin pharmacokinetics [16].

### Sleep measurement

Nocturnal sleep was evaluated using the bispectral index (BIS) (BIS XP, Quattro sensor; Aspect Medical Systems, Inc., Norwood, MA, USA), a signal-processing technique based on the electroencephalogram (EEG) previously used to evaluate sleep in critical care patients [17]. BIS data were recorded in 5-second intervals and downloaded onto a personal computer. Two outcome measures were used: sleep efficiency index (SEI) and area under the curve (AUC). SEI was defined as the ratio of a patient's total sleep time over the time available for 'nocturnal' sleep (9 hours, from 10 p.m. to 7 a.m., corresponding to nursing staff shift patterns). Sleep was defined as BIS below 80 [18]. AUC was calculated using the trapezoidal rule, which uses trapeziums to approximate the region under a curve and calculate its area. For each night, SEI and AUC values were set to missing if recordings were missing for more than 2 hours. Analyses were limited to nights 3 and 4 since the potential chronohypnotic benefits of melatonin are not immediate and may take 3 days to be realised [19,20]. All four nights were considered in a secondary analysis.

During the study, other sleep measurement methods were used with the main aim of evaluating agreement and comparing feasibility and reliability in the critical care setting. These included actigraphy (Actiwatch; Cambridge Neurotechnology Ltd., Cambridge, UK), nurse assessment (direct nurse observation using hourly epochs), and patient assessment (Richards Campbell Sleep Questionnaire [RCSQ]). Details of the methods and results on measurement agreement are reported elsewhere [21]. Results of these methods for the melatonin effect, expressed in terms of SEI, are reported here as secondary analyses.

### Statistical analysis

Differences between treatment groups in mean values of SEI and AUC, averaged over nights 3 and 4, were analysed using the *t *test with equal variances. For the secondary analysis, including all four nights, we used a multilevel model, Prais regression, which accounts for the within-patient correlation between measurements on successive nights. Mean and standard deviation (SD) or median and interquartile range were used as appropriate for descriptive statistics. The Pearson correlation was used for test of association. Data were analysed using Stata 9.1 software (StataCorp LP, College Station, TX, USA).

A sample size of 34 patients was calculated based on BIS SEI, assuming α = 0.05, power = 0.8, and minimum detectable difference in SEI = 0.20. Since no data on the SD of BIS SEI in critical care patients were available, we used the SD of SEI obtained using polysomnography as a proxy. Polysomnography studies reported SD values from 0.1 to 0.24 [2,22-24] and we used a conservative value of 0.20.

### Pharmacokinetic analysis

Pharmacokinetic analysis of plasma melatonin concentrations was undertaken in the first nine patients in the melatonin group. Twelve blood samples were collected from each patient at appropriately spaced intervals after the first oral dose. All samples were taken from the arterial line, immediately centrifuged, and stored at -20°C until assay. Plasma melatonin was measured in duplicate using a melatonin direct radioimmunoassay (Immuno Biological Laboratories, Hamburg, Germany). Sample dilution to within the linear range of the assay was undertaken as necessary. The values of intra-assay precision (percentage coefficient of variation) at plasma concentrations of approximately 10 and 150 pg/mL were 13.6% and 6.8%, respectively. The interassay coefficient of variation was 24.5%. Plasma concentrations were corrected for endogenous plasma melatonin concentration by subtracting the 9 p.m. baseline value. Non-compartmental pharmacokinetic analysis was undertaken (PKSolution 2.0; Summit Research Services, Montrose, CO, USA).

## Results

**Figure 1:**
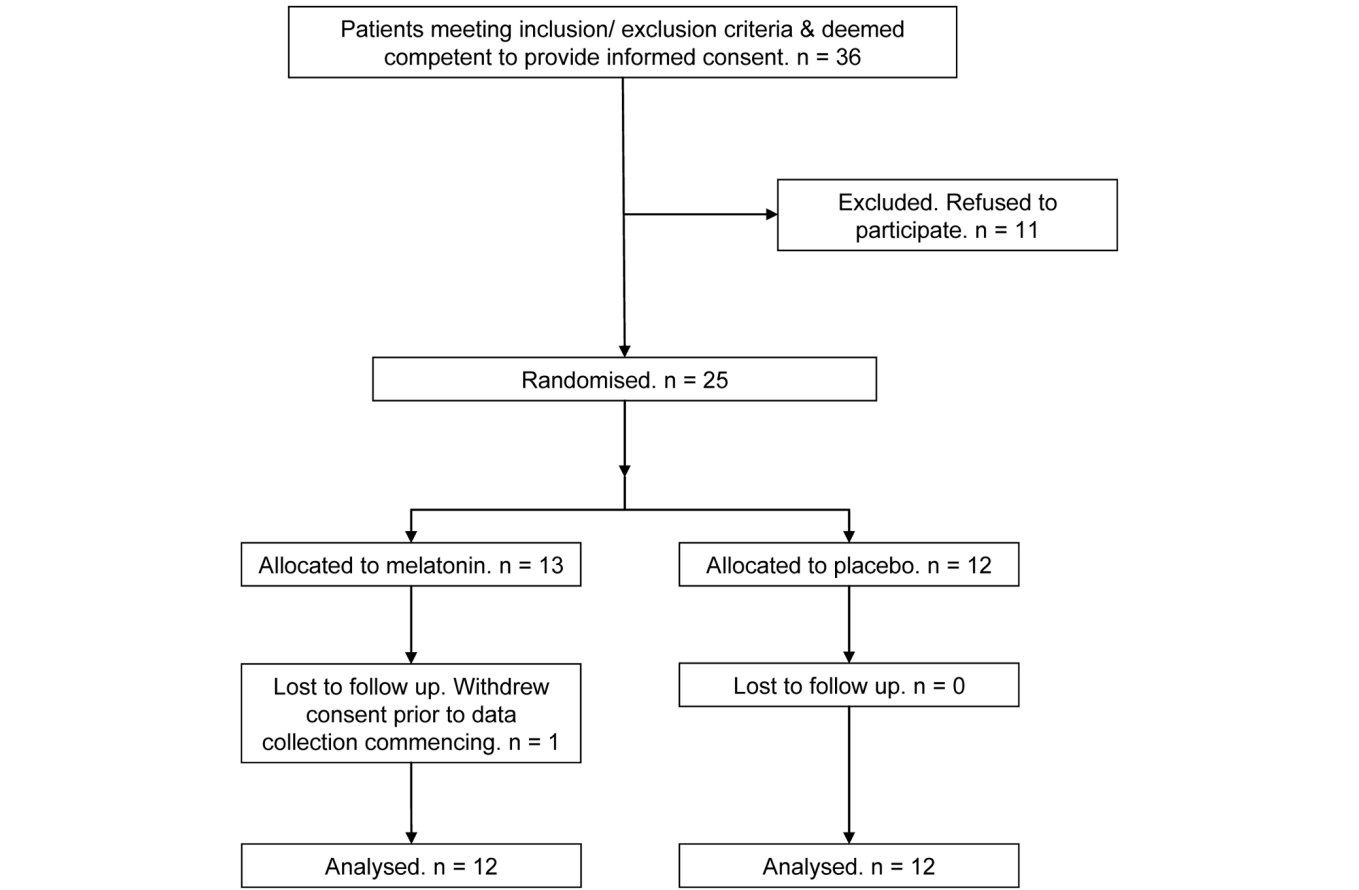
Flowchart of the study, from patient recruitment to analysis.

Figure 1 shows patients' inclusion in the study. Due to slow recruitment, we could recruit only 24 patients. There were 4 patients (3 in the placebo and 1 in the melatonin group) with missing data for nights 3 and 4, the reasons being discharged/re-sedated (4 nights), patient removed sensor (2 nights), signal quality index low (1 night), and patient refused (1 night).

Table 1 shows patients' baseline characteristics in the two treatment groups. An imbalance of known risk factors for sleep disturbances was present due to small sample size, potentially leading to more sleep disturbance in the melatonin group. Such factors included older age [25], delirium [26], and ventilation with pressure support ventilation (because of the possibility of desynchrony) [27]. No differences between the melatonin and control groups were observed with regard to either patient uptake of earplugs or eye masks (9% and 2% of nights, respectively) or nocturnal environmental disturbances score. The mean (SD) baseline illuminance at the head of each bed when all lights were turned off was 9.6 (2.6) lux.

**Table 1:** Baseline patient characteristics

Characteristic	Placebo (n = 12)	Melatonin (n = 12)
Male, number (percentage)	7 (58.3)	4 (33.3)
Reason for ICU admission, number (percentage)		
Severe sepsis	8 (66.7)	10 (83.3)
Postoperative respiratory failure	2 (16.7)	1 (8.3)
Pneumonia	2 (16.7)	1 (8.3)
Age in years, mean (SD)	58.7 (12.5)	69.9 (12.0)
APACHE II score on study entry, mean (SD)	16.8 (3.4)	17.3 (3.8)
Actual body weight in kilograms, median (IQR)	69.0 (57.4; 77.5)	65.0 (63.5; 70.0)
Ideal body weight in kilograms, mean (SD)	60.0 (6.9)	57.2 (6.5)
Body mass index, mean (SD)	24.6 (4.7)	25.0 (3.1)
Patients' usual sleep quantity in hours^a^, mean (SD)	6.5 (1.57)	6.2 (2.07)
ICU length of stay prior to study in days, median (IQR)	16.5 (13.0; 20.5)	16.5 (11.0; 19.0)
Time of ventilation prior to study in days, mean (SD)	20.0 (14.3)	13.6 (6.5)
Sedation (morphine/midazolam) prior to study, number (percentage)	2 (16.7)	2 (16.7)
Time since sedation stopped prior to study in days, mean (SD)	6.6 (2.9)	7.5 (4.7)
Delirium during study period, number (percentage)	1 (8.3)	4 (33.3)
Ventilation mode on nights 3 and 4, number (percentage)		
BiPAP/CPAP-ASB	7 (70.0)	7 (58.3)
External CPAP/Hi-flow oxygen	3 (30.0)	5 (41.7)

^a^Usual sleep time at home as reported by the patient. APACHE II, Acute Physiological and Chronic Health Evaluation II; BiPAP, biphasic positive airway pressure; CPAP, continuous positive airway pressure; CPAP-ASB, continuous positive airway pressure with assisted spontaneous breathing; ICU, intensive care unit; IQR, interquartile range; SD, standard deviation.

There was no disparity between the groups in their exposure to the number of potentially sleep-disruptive medications. In patients who received morphine and midazolam, sufficient time elapsed between discontinuation of sedation and study enrolment to limit the potential distortion of results due to accumulation of these agents. None of the patients received haloperidol on nights 3 or 4. Nocturnal sleep time did not seem to correlate with patients' severity of illness, as measured by APACHE II (Acute Physiological and Chronic Health Evaluation II) daily score, although the wide confidence interval does not allow us to draw definitive conclusions (*r *= 0.10; -0.36 to 0.52; *P *= 0.68).

Results of the effect of melatonin on primary and secondary sleep measurements are shown in Table 2. Nocturnal sleep time was 2.5 hours in the placebo group and was 1 hour longer in the melatonin group, although the difference was not statistically significant (Table 2). BIS AUC showed a statistically significant 7% decrease in the melatonin group, with lower AUC meaning 'better' sleep (AUC difference = -54.23; -104.47 to -3.98; *P *= 0.04). To account for the imbalance in baseline characteristics, we adjusted the analyses using linear regression. The small sample size limited the number of covariates we could adjust for [28] and we thus created a single variable indicating the overall baseline risk of sleep disturbances. High risk was defined as the presence of any two of the following: age of greater than or equal to 70 years, delirium positive, and ventilation with BiPAP (biphasic positive airway pressure) or CPAP-ASB (continuous positive airway pressure with assisted spontaneous breathing). The results of the adjusted analysis did not vary substantially, apart from an expected loss in precision of the estimates: SEI difference = 0.12 (-0.04 to 0.28; *P *= 0.12) and AUC difference = -48.76 (-103.06 to 5.54; *P *= 0.07). Any evidence of a treatment effect nearly disappeared when considering all four nights: SEI difference = 0.05 (-0.07 to 0.17) and AUC difference = -26.62 (-70.51 to 17.28). Results from the additional sleep measurement methods did not support those obtained with BIS and indeed were all inconclusive (Table 2). As regards possible side effects of melatonin, one patient in the melatonin group reported a headache on a single night, which responded to acetaminophen administration.

**Table 2:** Effect of melatonin on nocturnal sleep efficiency on nights 3 and 4, using different outcome measures

	Bispectral index sleep efficiency index (95% confidence interval)			
	
Sleep measurement method	Placebo group	Melatonin group	Difference	*P *value of the difference
Primary analysis				
Bispectral index	0.26 (0.17 to 0.36)	0.39 (0.27 to 0.51)	0.12 (-0.02 to 0.27)	0.09
Secondary analysis				
Actigraphy	0.75 (0.67 to 0.83)	0.73 (0.53 to 0.93)	-0.02 (-0.24 to 0.20)	0.84
Nurse assessment	0.51 (0.35 to 0.68)	0.45 (0.26 to 0.64)	-0.06 (-0.29 to 0.17)	0.58
Patient assessment (RCSQ)	0.50 (0.43 to 0.58)	0.41 (0.24 to 0.59)	-0.09 (-0.28 to 0.09)	0.32

RCSQ, Richards Campbell Sleep Questionnaire.

**Figure 2:**
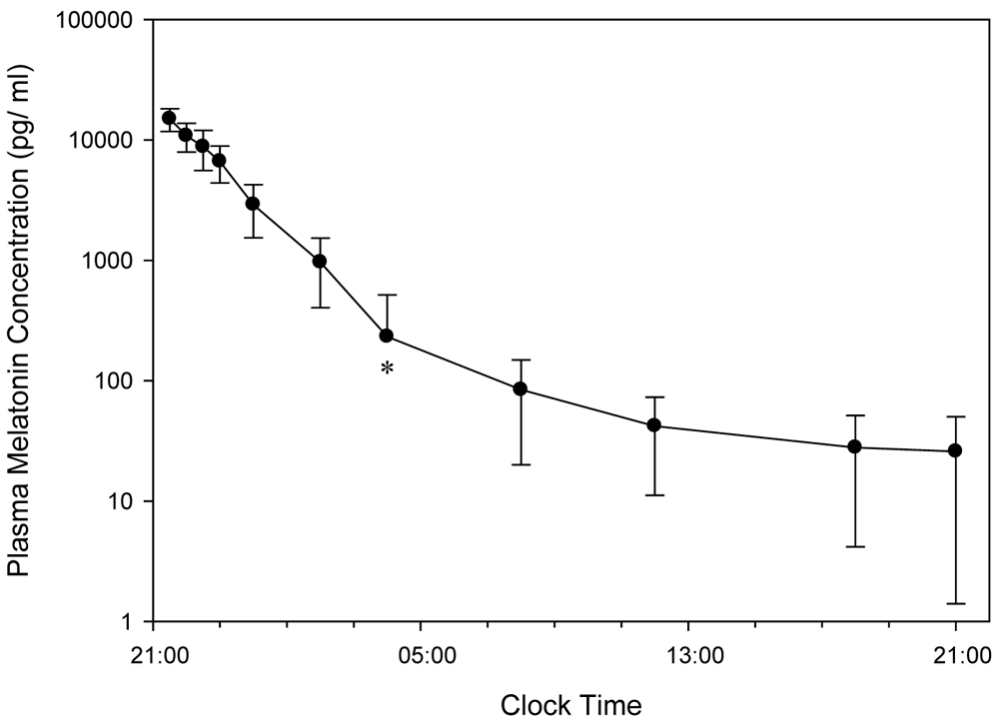
Semi-logarithmic plot of mean melatonin plasma concentration (± standard deviation [± SD]) versus clock time after a 10-mg oral solution dose administered at 9 p.m. in critical care patients. *4 a.m. data point. Mean concentration value minus SD is a negative number and cannot be represented on a logarithmic scale.

The main pharmacokinetic data are summarised in Table 3. Plasma melatonin concentrations declined bi-exponentially (Figure 2). Both maximum plasma concentration (C_max_) and AUC_(0–24) _(area under the concentration time curve between time 0 and 24 hours) had a moderately strong correlation with plasma alanine transaminase concentrations (*r *= 0.70; 0.06 to 0.93; *P *= 0.04, and *r *= 0.62; -0.07 to 0.91; *P *= 0.07, respectively). No such association was found with age, gender, weight, creatinine, or bilirubin. No association was found between the pharmacokinetic parameters: C_max_, AUC_(0–24) _or C_(08) _(plasma concentration at 8 a.m.), and mean SEI or BIS AUC measurements of nocturnal sleep.

**Table 3:** Summary of main pharmacokinetic results

T_max_, hours	C_max_, pg/mL	AUC_(0–24)_, ng-hours/L	Overall t_1/2_, hours	Oral clearance (Cl/F), L/hour	C_(08)_, pg/mL
0.5 (0)	14,974 (3,200)	29,979 (8,205)	1.47 (0.28)	351.0 (96.7)	84 (64)

Data are presented as mean (standard deviation). AUC_(0–24)_, area under the concentration time curve between time 0 and 24 hours; C_(08)_, plasma concentration at 8 a.m.; Cl/F, clearance/bioavailability (oral dose/area under the zero moment curve); C_max_, maximum plasma concentration; t_1/2_, plasma half-life; T_max_.

## Discussion

Our study confirms previous findings [17,29] that nocturnal sleep in patients being weaned from mechanical ventilation is highly compromised, with an average of only 2.5 hours in the placebo group. Melatonin therapy was associated with a 1-hour increase in nocturnal sleep compared with placebo, corresponding to an increase of 47%, although the SEI difference did not reach statistical significance. We found a statistically significant reduction of 7% in BIS AUC with melatonin administration, suggesting sleep improvement. The use of AUC has some advantages compared with SEI. Apart from providing greater statistical power, BIS AUC provides an indication of both sleep quantity and quality [17], which might be more informative than sleep quantity alone. However, the clinical significance and interpretation of a reduced AUC remain unclear [30].

Two other small trials investigated the effect of melatonin on nocturnal sleep in critically ill patients [11,31], but comparison is limited due to the use of different sleep measurement methods, for which agreement is rather poor [21]. In fact, although polysomnography is the gold standard for quantifying and qualifying sleep, the challenges of the critical care environment have led to the use of a number of alternative methods [21]. The first study was a crossover trial that used actigraphy on eight respiratory patients and showed positive results [31]. Baseline sleep was reported to increase from approximately 3 to 6 hours with melatonin administration, although results of the comparison between melatonin and placebo were not reported. The second study used nurse observation to evaluate 32 tracheostomised patients and showed negative results [11]. Placebo patients slept for about 4 hours, with only 15 minutes more in the melatonin group. As a measure of sleep, actigraphy is not ideal in critically ill patients, being influenced by abnormalities of the neuromuscular system which are common in these patients [21]. As regards nurse observation, intensive observation of sleep (5-minute intervals) is probably necessary to allow differentiation between interventions in critical care studies [32] and even then it suffers from being a subjective measure that may overestimate sleep quantity [33]. Patient assessment has been used in critical care sleep studies on other interventions but its applicability is limited by patients' acute cognitive and perceptual problems [21]. We chose to use BIS as the primary outcome measure since it provides an objective measure of sleep which is not adversely affected by the presence of neuromuscular weakness. However, the BIS, similar to other EEG-based techniques, can be adversely affected by conditions such as traumatic brain injury, dementia, or delirium which result in EEG slowing [21]. Although we used BIS XP technology, a degree of susceptibility to increased BIS values as a consequence of electromyogram artefact remains [34]. In our study, the results from actigraphy, nurse observation, and patient assessment, which we used as secondary outcome measures, were all inconclusive. Differences between our BIS SEI results and those of our other measures may be explained somewhat by residual neuromuscular weakness in patients recovering from sepsis (actigraphy), the use of hourly epochs (nurse assessment), and limitations in the patients' ability to complete the RCSQ (patient assessment) [21], all of which may lead to overestimates of sleep quantity and SEI.

Melatonin appears to have a favourable adverse effect profile; headaches, dizziness, nausea, and drowsiness are the most common adverse events reported with short-term melatonin administration [35]. Melatonin treatment appeared to be well tolerated in our patients, with only one patient reporting a single episode of headache.

Melatonin appeared to be rapidly absorbed from the oral solution, and peak concentrations were higher than those reported for comparable doses in healthy individuals [36,37]. After oral dosing, the C_max _is affected by the solubility of melatonin in the formulation, alterations in bioavailability, and clearance. Orally administered melatonin is subject to an extensive 'first-pass effect', with bioavailability reported to be approximately 15% [38], although there is high variability due to factors such as cytochrome P450 1A2 (CYP1A2) activity and co-administration of interacting drugs [39]. The acute inflammatory cascade related to sepsis may adversely affect cytochrome P450 regulation, including CYP1A2 enzyme activity [40,41], and a prolonged reduction in enzyme function in patients recovering from critical illness may have contributed to the high peak concentrations. Conversely, the high C_max _and AUC_(0–24) _could not be accounted for by concurrent use of CYP1A2 inhibitors. Although conventional liver function tests are poor predictors of hepatic drug metabolism, there was a moderate correlation between plasma transaminase levels and measures of exogenous melatonin exposure. Contrary to a report of endogenous plasma levels in cirrhotic patients [42], no such association was found for total bilirubin, although the power of our analysis was limited.

We also found no association between markers of drug exposure and nocturnal sleep quantity. The soporific and entraining effects of melatonin have been shown to reach a plateau at plasma concentrations lower than those described in our patients [43]. Therefore, having plasma concentrations in excess of the dose-dependent range would not be expected to demonstrate further improvements in sleep efficiency. The ideal dosing schedule of melatonin would produce an appropriate rapid peak plasma concentration while maintaining 'physiological' plasma levels over the nocturnal period. Our patients were unable to receive a modified release formulation, being fed via enteral feeding tubes, and hence we used a relatively large immediate-release formulation to ensure continuous nocturnal exposure. As described by others [44], the administered dose resulted in some patients with relatively low clearance having potentially 'nocturnal' plasma levels during the late morning. This may have negated some of the potential chronotherapeutic benefits of melatonin [12]. The presence of supraphysiological levels in the morning will have a phase-delaying effect and thereby negate some of the benefits of the phase-advancing effect of the 9 p.m. administration. However, we did not find an inverse correlation between nocturnal sleep markers and melatonin plasma concentration at 8 a.m. as might therefore be expected. Our pharmacokinetic data suggest that immediate-release doses of 1 to 2 mg administered at 9 a.m. might provide suitable nocturnal plasma melatonin concentrations whilst minimising the risk of daytime overdose.

### Limitations of the study and suggestions for future research

There are a number of obvious limitations in our study which should be reviewed when considering the methodology of future studies. The study was smaller than planned, with only 71% of the target sample size being reached, mainly due to problems in obtaining consent in the most acutely ill patients. Statistical power was further decreased because of missing data. Both of these problems should be taken into account when designing a study, particularly in deciding on the inclusion criteria and complexity of the study protocol. The small sample size also meant that we had imbalances in baseline characteristics between the groups, although our attempt to adjust for important sleep-related factors (age, delirium, and ventilator status) did not materially alter the results.

Our use of alternative sleep measurement techniques to polysomnography also limited the scope of our results. We did not have sleep-stage data and therefore cannot comment on the effect of melatonin on SWS or REM sleep phases. The ultimate aim of sleep interventions in critical care patients is to attempt to consolidate nocturnal sleep and increase both SWS and REM sleep phases. At low doses, melatonin has a sleep-promoting effect without a significant adverse effect on normal sleep architecture [45], a potential advantage over conventional hypnotic agents. Indeed, it could be suggested that the improvements in sleep quantity observed may have been achieved with a conventional hypnotic agent (for example, zopiclone). The significant potential for adverse cognitive effects of these agents, particularly in older patients [46], still makes melatonin (or melatonin agonists such as ramelteon) worth continued investigation.

Ideally, polysomnography should be used as a continuous measure of sleep in further studies. However, such an application presents significant logistical and technical challenges and is associated with specific difficulties, including patient tolerability and sleep-stage interpretation in patients experiencing complex electrophysiological changes [17].

We did not have a useful measure of daytime sleep because our actigraphy data significantly overestimated nocturnal and diurnal sleep quantity [21] and our BIS recording was restricted to the nocturnal period due to patient tolerability. We are therefore unable to comment on the effect of melatonin on daytime sleep. While we are primarily interested in optimising nocturnal sleep with interventions, we should not ignore the potential impact that diurnal sleep periods have on nocturnal sleep efficiency. Approximately half of total sleep time of critical care patients may occur during the diurnal period, with significant inter- and intra-patient variability as to whether sleep deprivation is present over 24 hours [3].

Our environmental score provided only a guide to nocturnal patient disturbances. Noise, light, and patient disturbances have been shown to account for approximately 30% of nocturnal arousals and awakenings [47]. Although the ambient nocturnal illuminations were at an appropriate level to allow normal melatonin secretion [48], we did not have an accurate measure of light interruptions. The absence of continuous light and noise measurements and lack of quantification of patient disturbances by staff are therefore further potential limitations. Earplugs can improve sleep quality in healthy volunteers exposed to simulated intensive care noise [49]. However, we found that patient willingness to use eye masks and/or earplugs was very low, which limits their routine clinical application. Finally, future studies should consider extending the sleep intervention to a coordinated bright light and exogenous melatonin therapy. The sleep-wake process relies on a combination of homeostatic and circadian factors for its optimum function [50], and the full activity of melatonin on the sleep-wake cycle in humans requires the coordination of other time cues such as light [12].

## Conclusion

Although suggesting a possible future role of melatonin in the routine care of critically ill patients, our findings need to be confirmed by a larger, possibly multicentre, randomised controlled trial, ideally using polysomnography as a continuous measure of sleep quantity and quality. A 10-mg nocturnal dose of melatonin is excessive in this patient population and reduced doses of 1 to 2 mg could be used in future chronotherapeutic studies.

## Key messages

• Nocturnal sleep quantity in patients being weaned from mechanical ventilation is highly compromised.

• Melatonin therapy may increase nocturnal sleep quantity, but further investigation using continuous polysomnography is necessary to provide sleep quality information.

• A 10-mg dose of melatonin produces supraphysiological morning plasma levels in critical care patients, possibly negating some of the phase-advancing effects of nocturnal administration.

• Immediate-release doses of 1 to 2 mg administered at 9 p.m. might provide suitable nocturnal plasma melatonin concentrations whilst minimising the risk of daytime overdose.

## Abbreviations

AUC = area under the curve; AUC_(0–24) _= area under the concentration time curve between time 0 and 24 hours; BIS = bispectral index; C_max _= maximum plasma concentration; CYP1A2 = cytochrome P450 1A2; EEG = electroencephalogram; ICU = intensive care unit; RCSQ = Richards Campbell Sleep Questionnaire; REM = rapid eye movement; SAS = Sedation Agitation Scale; SD = standard deviation; SEI = sleep efficiency index; SWS = slow-wave sleep.

## Competing interests

The authors declare that they have no competing interests.

## Authors' contributions

RSB conceived the clinical study, enrolled patients, collated the data, and analysed and interpreted the results. GHM participated in the design of the clinical study and the data analysis. CM completed the statistical analysis and assisted with the interpretation of results. All authors contributed to, read, and approved the final manuscript.

## Supplementary Material

Additional file 1 Environmental disturbances log Click here for file
