# Supplementary material for: Melatonin therapy to improve nocturnal sleep in critically ill patients: encouraging results from a small randomised controlled trial
Source: Crit Care. 2008 Apr 18;12(2):R52. doi: 10.1186/cc6871 (PMC2447606; doi:10.1186/cc6871)
Supplement: Additional file 1 — Environmental disturbances log [file cc6871-S1.doc]

**Additional file**

Study Patient No: Nurse Initials:

Day No: Bed No:

**Please circle any of the following environmental disturbances occurring between 22:00 and 07:00**

1. Were any of the ITU lights identified as >100 lux (marked with red sticker on switch) switched on between 22:00 and 07:00?

YES NO^0^

1. **If YES, how frequently did this occur?**

###### 1-3^1^ 4-7^2^ >7^3^

1. **Was the study patient disturbed for clinical reasons by medical or nursing staff?**

YES^1^ NO^0^

1. **Was the patient adjacent to the study patient disturbed for clinical reasons by medical or nursing staff during the study period?**

YES^1^ NO^0^

1. **Did any of the following occur on the ICU during the study period?**

Patient Death^1^ Resuscitation^1^

Patient Admission^1^ Patient Transfer Out/ Bed change^1^

**Total Environmental Score =**

**How would you rank the noise level last night (compared to ‘normal’)?**

Quieter than normal Average Noisier than normal
